# Two-dimensional phonon engineering triggers microscale thermal functionalities

**DOI:** 10.1093/nsr/nwz114

**Published:** 2019-08-08

**Authors:** Run Hu, Xiaobing Luo

**Affiliations:** State Key Laboratory of Coal Combustion, School of Energy and Power Engineering, Huazhong University of Science and Technology, China

Thermal metamaterials enable heat-flow manipulation alongside many emerging thermal functionalities. However, macroscale experimental performances are usually less satisfactory due to the errors of effective medium design, imprecision fabrication and the non-negligible thermal-contact resistance. In the *Nano Letters* paper [1], Wu and coworkers propose the ion-write micro-thermotics (IWMT) platform, on which they overcome the issues in the macroscale and experimentally demonstrate the microscale thermal cloak and rectifier functionalities. The fundamental progress is the successful experimental demonstration of two-dimensional (2D) phonon engineering, which can be applied to explore more microscale thermal functionalities and reveal more physics with newly-added spatial phonon-distribution information. To highlight the progress, this perspec-tive shares the status, challenges and potential directions to facilitate discussion and research on thermal metamaterials and phonon engineering.

Molding heat flow has long been considered impossible, or at least formidable, due to its diffusive nature, which is governed by the Laplacian-type equations, not akin to the parabolic-wave equations that have been successfully applied to manipulate the electromagnetic waves, light and sound with great advances in related technologies and convenience in our daily life. But this long-term challenge is eased with the birth of thermal metamaterials in recent decades [2,3]. Based on thermal metamaterials, many novel and fascinating functionalities are proposed and demonstrated theoretically and experimentally, including, but not limited to, thermal cloak, concentrator, illusion, camouflage, refraction, reflection, encoding, diode, printing, etc. [2–6]. Comparably, thermal cloak is the most attractive, in which heat flow is bent around and returns to the original direction as if the heat flows through a homogeneous plate. As a result, any objects sitting in the cloaking region can be insulated from the outside temperature change and remain at uniform temperature if the objects generate no heat, being rendered invisible from the outside observers.

Although the theoretical demonstrations of macroscale thermal-cloaking performance are usually perfect, the experimental ones are barely satisfactory. For the practical fabrication of thermal metamaterials, including thermal cloaks, the effective medium approximation theory is frequently used to design the alternating layers of two kinds of materials with distinct thermal conductivities like metal and polymers, or to design the number, size and distribution of the drilled strips or holes in a metal plate [4,5]. Note that such fabrication alternatives with the nature-existing materials, though barely solvable and feasible, are not accurate enough to achieve satisfactory cloaking performance. More-over, the mismatched thermal expansion and thermal interfacial resistances between the layered structures are usually neglected for both design and evaluation.

**Figure 1.**
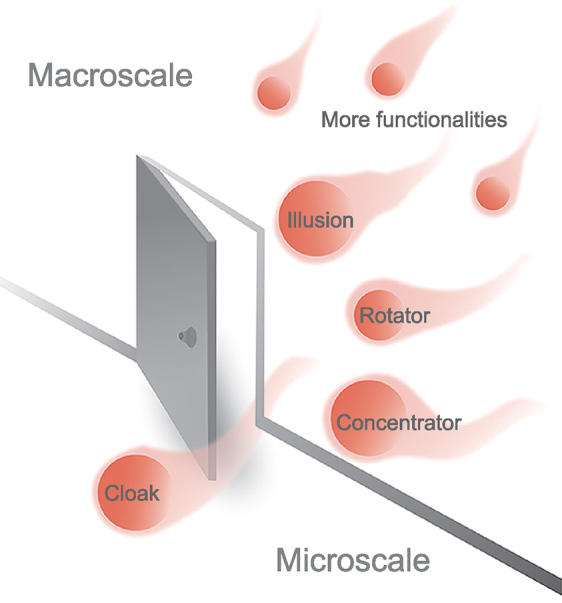
Schematic for the thermal functionalities from macroscale to microscale.

Experimentally, the paper by Wu and coworkers in *Nano Letters* develops a platform for IWMT that can perfectly solve the above-mentioned experimental issues for macroscale thermal cloaks and even other thermal functionalities [1]. Their idea is exactly inspired by the pending issues in the macroscale experiments on thermal cloaking and advances in three aspects: (i) they continuously tune the local thermal conductivity of 120-nm-thick silicon membrane from the crystalline value to the amorphous value by adjusting the irradiation dose of a helium ion microscope; (ii) no exotic materials (atoms) are added into the background plate (silicon membrane), thus the troublesome thermal-contact resistance between different materials and structures is totally removed; (iii) a significant advance in their experiment is that they use the thermoreflectance imaging technique to map the microscale temperature field, enabling the microscale thermal-cloaking demonstration. Bene-fiting from these advances, the paper has garnered great interest and also demonstrates the feasibility for molding heat flow at the microscale beyond the functionalities like thermal cloaking, rectifying and management.

In essence, their IWMT is a platform for 2D phonon engineering. Most of the existing phonon engineering only deals with the ‘quasi-one-dimensional (quasi-1D)' or ‘end-to-end' phonon-transport properties, like phonon transmission and effective thermal conductivity. The 2D spatial distribution of phonon energy has largely stayed in the theoretical work [7]. Here, the continuous tuning of local thermal conductivity stems from phonon scattering by point defects and amorphous domains generated by helium ion irradiation at different doses on the 2D silicon membrane. The spatial resolution, corresponding to 2D phonon engineering, can be as high as ∼ 10 nm, which is much smaller than the phonon mean free path in crystalline silicon. It is significant progress on phonon engineering, which enables a higher degree of freedom for next-level microscale heat-flow manipulation.

Based on 2D phonon engineering, more work can be done experimentally. (i) Exploring the particle nature of phonons experimentally. In the last two decades, much progress has been achieved by viewing phonons as incoherent particles due to their relatively small wavelengths and broad spectra of phonon population [7–10]. The experimental 2D distribution of incoherent phonon particles will be fairly interesting, which can be further compared with the Monte Carlo or molecular dynamics simulations revealing more physics, like local phonon scattering. (ii) Exploring the wave nature of phonons experimentally. Due to the strong scattering characteristics of phonons, their wave nature has long been neglected, to which, nevertheless, importance has begun to be attached in recent decades, since the particle-based predictions overestimate the experimental lattice thermal conductivities in superlattices and silicon nanomehes [9,10]. Previous work on exploring phonon-wave nature mainly remains in quais-1D superlattices and wave nature is only discussed in terms of the tuning of thermal conductivity [8–10]. The 2D phonon engineering can be used to show the spatial map of coherent phonon waves experimentally, which can be further validated with the phonon-wave packet and atomistic Green's function simulations. (iii) Exploring more physics of phonon transport. Physics in terms of phonon transport includes phonon scattering, phonon localization, wave-particle duality, constructive/destructive interference, etc. The 2D phonon-engineering-based phonon landscape provides additional information and enables exploring new physics, like local phonon localization, local phonon interference, spatial/temporal modulation of phonons, etc. (iv) More naturally, in view of the successful demonstration of thermal cloaks at the microscale with 2D phonon engineering, it would be interesting to evaluate whether such an IWMT platform can be applied to achieve other microscale thermal functionalities. The challenge of anisotropic and inhomogeneous material properties in transformed thermotics has been avoided in their work. But, actually, the continuous tunability of local thermal conductivity of the silicon membrane shows great potential to achieve anisotropic and inhomogeneous thermal conductivities, which should be further examined first. The finer structural features in the quadlayer cloak with tuned local thermal conductivity have replenished us with confidence. Once validated, the microscale counterparts of concentrator, rotator, illusion, etc. can be explored successively if only these thermal-functionality counterparts are necessary and useful at the microscale. Besides exploring new physics, 2D phonon engineering can be also used to tune and shield phonon transport in nanoelectronics and nanophotonics, since efficient dissipation of heat in these nanoelectromechanical systems is challenging. Another potential application is thermal memory, as demonstrated on their reversible and rewritable IWMT platform [1].

It is quite inspiring to explore these possible microscale thermal functionalities with 2D phonon engineering. It is a new and advanced technique, which may offer the additional degrees of freedom to tune phonon transport with revealing new physics. We hope the paper by Wu and this perspective can stimulate greater interests and attentions on exploring microscale thermal functionalities and promising applications of 2D phonon engineering, which will enrich the understanding and broaden the boundaries of heat-flow manipulation from the macroscale to the microscale. Just as shown in Fig. 1, the door has been opened, and let us see.
